# 

**Published:** 1845-04

**Authors:** 


### CONTENTS

OF NO. IV.

### ORIGINAL COMMUNICATIONS.

### ESSAYS AND CASES.

ART. I.—Lectures on Diseases of the Chest. By J. R. BUCK, M.D., Lecturer on the Theory and Practice of Medicine in the Louisville Summer School of Medicine, - - - - - - - - - - - - 277

ART. II.—Remarks on the Importance of Good Health in Ministers of the Gospel, and on the best means of attaining it. By WM. M. BOLING, M.D., of Montgomery, Alabama. - - - - - - - 283

### REVIEWS.

ART. III.—Remarks on the Influence of Mental Cultivation and Mental Excitement upon Health. By AMARIAH BRIGHAM, M.D., Superintendent and Physician of the State Lunatic Asylum, Utica, N. Y. - - - 314

ART. IV.—The Principles of Surgery. By JAMES MILLER, F.R.S.E., F.R.C.S.E., Professor of Surgery in the University of Edinburgh, Surgeon to the Royal Infirmary, &c. - - - - - - - - - 330

### SELECTIONS FROM AMERICAN AND FOREIGN JOURNALS

Rheumatism, - - - - - - - - - - - - - 337

Magnesia, - - - - - - - - - - - - - - 339

Origin of Fat in the Animal Body, - - - - - - - 341

Phlegmasia Dolens, Gonorrhœa, and Rheumatism, - - - 342

Medical Society of London, - - - - - - - 342
A Case in which 16 Sewing-Needles and 4 Pins were extracted from the Wrist, - - - - - - - 345
Case of Sudden Blindness, - - - - - - - 346
Comparison between a new Vaccine Virus and that of 1836. 347
Statistics of the Medical Practitioners of Paris, - - - 347
An easy and certain method of performing Catheterism, - 348
Baron Larry's Medical Campaigns, - - - - - 349
Treatment of Itch in Belgium, - - - - - - 357
Pills of Iodide of Iron, - - - - - - - 357
Squill, - - - - - - - - - - - 358
Artificial Dilatation of the Os Uteri, - - - - - 359

ORIGINAL INTELLIGENCE.

Lectures on Geology, - - - - - - - 361
Commencement in the Medical Institute, - - - - 362
Mesmerism, - - - - - - - - - 362
Somerby's Concentrated Blow-pipe and Furnace, - - 363
New York Journal of Medicine, - - - - - - 364
Quack Advertisements, - - - - - - - - 364
St. Louis Medical and Surgical Journal, - - - - 365
British and Foreign Medical Review, - - - - - 365
Christison on Poisons, - - - - - - - - 366
Professor Paine's Introductory Lecture, - - - - 366
Medical Classes, - - - - - - - - 367
Books Received, - - - - - - - - 368
Louisville Summer School of Medicine, - - - - 368

